# Human-centred health-care environments: a new framework for biophilic design

**DOI:** 10.3389/fmedt.2023.1219897

**Published:** 2023-07-25

**Authors:** Bekir Huseyin Tekin, Rosa Urbano Gutiérrez

**Affiliations:** School of Architecture, University of Liverpool, Liverpool, United Kingdom

**Keywords:** biophilic design, therapeutic environment, non-clinical environment, cancer, nature

## Abstract

Increasing research corroborates that the qualities of the setting in which a patient receives healthcare positively influence health outcomes. Therefore, it has become progressively important to review the concept of therapeutic environments, as places where patients are treated with the most advanced medicine and technology, but also support their users in psychological, emotional and social terms. This quest for the optimal healing environment brings to the forefront the need to include other parameters in our design briefs, where the application of biophilic design proves to be paramount, as exposure to nature is associated with multiple health benefits. However, current biophilic design frameworks fail to provide efficient guidance, as their design recommendations don’t differentiate the level of value of each design parameter for each building programme and context. Our position is that a biophilic design framework can only be efficient if it is adapted to specific building functions and is geographically and culturally contextualized. This study assessed the application of biophilic design in therapeutic environments for cancer patients in the UK, and provided a revised conceptual framework that can more efficiently guide designers and policies in future interventions. This framework was informed by synthesised analyses from healthcare environments on the user's experiences, and primary data obtained from semi-structured interviews with architects and managers, which was then benchmarked against scientific data about the impact of biophilic design on humans. This comprehensive approach helped to identify and rank those biophilic design parameters that appear the most critical for promoting and supporting health and wellbeing in cancer healthcare settings and provided an up-to-date compilation of crucial design actions to enact the necessary change in future research and design practice.

## 1. Introduction

In the UK, statistics show that three million people have cancer, and this number is projected to be 5.3 million by 2040 (1). Cancer treatment follows a combination of chemotherapy, radiotherapy and surgery, and the treatment process may take years where the patients have to attend clinics regularly (2, 3). Along with physical problems, the diagnosis of cancer also brings psychological and social problems (2, 4–7). Recent research showed that the majority of cancer patients reported that the mental health consequences of cancer diagnosis are worse than the physical effects of cancer (8). Indeed, about 30% of the patients face mental health problems during cancer treatment (4, 6, 7), where depression, anxiety, and adjustment disorders are the most often diagnosed conditions (2, 4, 9, 10). Depressed or anxious people have lower social functioning, more disabilities, and overall functional impairment than people who are not affected by these conditions. Stress and anxiety also cause other problems such as pain, fatigue, and sleeping disorders (2). As with other chronic illnesses, cancer can generate fear, anger, guilt, confusion, feelings of loss of control, and sadness (2, 11). Taste and smell changes are also frequently observed side effects of chemotherapy, which are reported by cancer patients as one of the most distressing side effects together with hair loss, sleeping difficulties, vomiting, loss of appetite, fatigue, nausea and change in temperature perception (12, 13).

All of these aspects can be supported through environmental design. Supporting evidence suggests that the environmental qualities of therapeutic settings impact users' health and well-being outcomes (14–20). The search for ideal healing environments has been gaining more attention in research practices since the 1950s. Provision of optimal healing environment theories (e.g., attention restoration theory, supportive design theory, therapeutic environment theory, stress recovery theory, salutogenesis) indicates the need to incorporate some parameters where the role of nature, and with it, the application of biophilic design, is essential (21, 22).

The emergence of biophilic design as a discipline refers to “*the innate human connection to nature and natural processes to promote health and well-being in the spaces we inhabit*” (23–25). Supporting evidence suggested that contact with nature is associated with many physiologic health benefits including faster recoveries, less medication, lower blood pressure, and pain reduction (26, 27). Moreover, exposure to nature and biophilic elements promotes emotional, spiritual and mental health, decreases stress and anxiety, and triggers positive shifts in mood (28–30) which are crucial for cancer patients since psychological distress, fatigue, anxiety or depression are among the main problems they confront (9, 16, 19, 31, 32).

Pioneers in this field formalised biophilic design frameworks. A first framework was presented in *Dimensions, Elements and Attributes of Biophilic Design: The Theory, Science and Practice of Bringing Buildings to Life* (25), which superficially examined the biophilic design parameters regardless of their applicability to design practice. Also, this framework did not specify any connections between parameters and building typology or did not demonstrate any evaluation of the relative value of the different parameters. Kellert's second framework, included in *The Practice of Biophilic Design* (23), provided a clearer organisation and focus, systematising the biophilic design parameters in a more comprehensive way with the aim to inform their application in practice. However, as with the previous framework, it lacked specificity and the hierarchisation of parameters for each type of building programme. A third framework, which is the most commonly used in research and practice, was described in *14 Patterns of Biophilic Design* (33), considering biophilic design parameters in an interdisciplinary context. This book was fundamental in providing a more comprehensive framework to define and assess design based on biophilic principles more precisely, using a classification supported by empirical evidence. By doing so, it aimed to be adaptable to the application or development of biophilic designs. It was approached to list scientifically supported recommendations to inform general design practice, but the parameters were not ranked by importance level for different building functions and contexts. Following this framework, a design guideline titled *Nature Inside: A Biophilic Design Guideline* (34), explained the economics and design steps for the biophilic design process and examined case studies of applied biophilic design regarding different building typologies: housing, schools, retail, offices, hotels, hospitals, factories, and communal spaces. However, the guideline was useful to provide successful examples but did not direct designers on a clear path to efficient biophilic design. Additionally, Salingaros (35) proposed a framework with a formulated index system which aimed to quantify the level of biophilia in buildings using objective data (factual data on the impact of biophilia on human health), but still, this proposed framework is not concerned with differentiating which parameters are more relevant for different building programmes and contexts (e.g., therapeutic environments).

Within this context, our position is that the current guidance is not efficient enough to support design practice. Biophilic design is not just about listing all the parameters that can put us in close proximity to nature, or about examining how to design with natural elements individually. Equally, the inclusion of natural elements *per se* is not exclusive to biophilic design, and the prescription and regulation of many of these parameters form part of every design guideline. Our view is that biophilic design can only be fully achieved if the relevant parameters for the targeted user groups work together harmoniously in the space. For example, designing a garden from a biophilic perspective cannot just be for providing oxygenised fresh air, but also for enabling a full experience in connection with nature, including a multi-sensory immersing scenario with all parameters working in synergy: it is about the greenery, water and animals, but also about the smells, freshness, colours, sounds, textures and the elicited feelings of mystery, prospect, refuge or even danger. This is what biophilia is about, feeling the connection with nature in that complex and intricate relationship between different parameters. The ideology that the above-mentioned pioneers presented did not focus on how to bring that complete connection to a design project. Moreover, not all parameters are equally important for every type of building as the activities we do are different and require different environmental conditions. This harmony should be established for each building typology (educational, commercial, industrial, therapeutic, etc.) and attend to climate and local culture since people have different notions and perceptions of nature in different regions. An optimised biophilic design framework should be specific to the building programme, user profile, and context.

Thus, this paper reports a multi-method study, whose main goal is the provision of a novel biophilic design framework, with a particular focus on non-clinical therapeutic environments for cancer patients. This research also aims to hierarchise the parameters included in the new framework in a way that can more efficiently guide designers, revealing which are the most critical for promoting and supporting human health and wellbeing in these environments.

## 2. Methodology

The methodological approach included data collection from two different sources: [1] Scientific evidence, and [2] Professional practice and experience. A narrative literature review, a systematically searched literature review, and a meta-synthesis analysis informed the scientific evidence using secondary sources, while professional practice and experience were investigated with primary data collected from semi-structured interviews. The separate findings from the meta-synthesis (Study 1) (36) and the systematically searched review (Study 2) (37) have been extensively discussed in two published articles, so this paper primarily focuses on how the outcomes of these two studies, together with the data obtained from the semi-structured interviews with practitioners (Study 3), generated the final framework as a design system.

**Figure 1:**
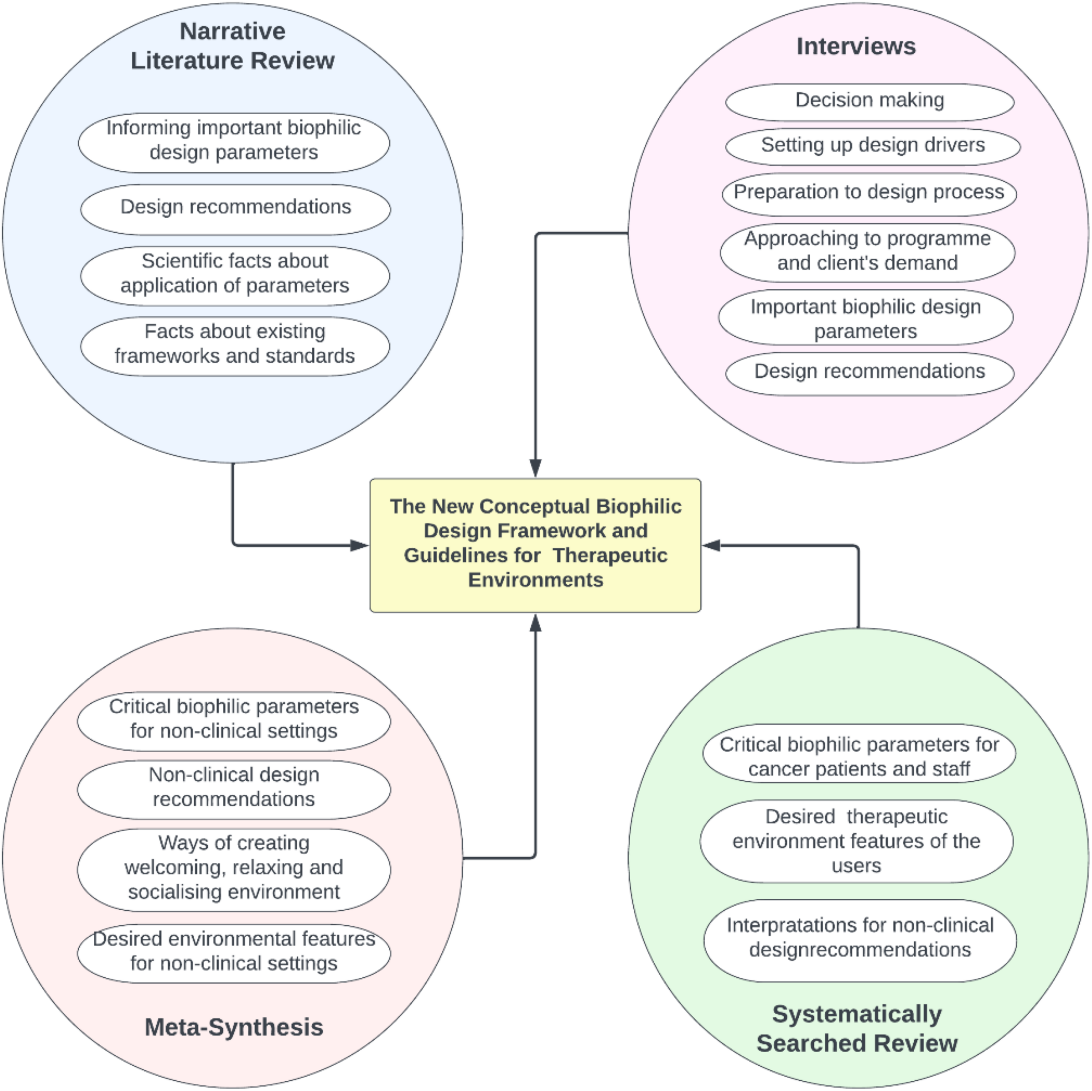
Data compilation map of the biophilic design framework.

The research focused on non-clinical therapeutic environments, taking Maggie's Centres as the main case study. These centres are paradigmatic examples of human-centred design, where nature and the connection between inside and outside play a major role in their briefs (38). The meta-synthesis and the interviews were mainly focused on these centres, so the obtained data directly contributed to the definition of the new framework for non-clinical therapeutic environments. The systematically searched review together with the narrative literature review fed the framework indirectly, since their data were extensively extracted from studies focused on clinical environments, but the patients' needs and statements gave benchmarks that could be translated into non-clinical environment design. Figure 1 depicts the process for the compilation and synthesis of data from each method.

### 2.1. Study 1 outcomes

The meta-synthesis of 13 studies about Maggie's Centres provided an extensive examination of users' comments on a broad range of design aspects of non-clinical therapeutic environments for cancer patients and gave an insight into the value of biophilic design for users. The comments from Maggie's users revealed essential information about how people understand, perceive and value nature and why biophilia is about all important biophilic parameters that should work together in harmony.

This study identified four groups of biophilic design parameters ranked in order of importance as follows: [1] Daylight, Greenery-Plants, Fresh Air*, [2] Sense of Belonging-Personal Past, Multisensory Environment, Refuge-Feeling Safe, View-Prospect, [3] Natural Material, Colour, Bringing the Outside to the Inside, Spaciousness, Curiosity and [4] Seasonal Changes, Thermal Comfort-Variability, Water, Fire. This ranking is completely based on the users' statements in these 13 studies, and how frequently they emphasized the importance of these parameters.

Apart from the provision of important biophilic design parameters in Maggie's centres, Study 1 proposed the following general guidelines regarding the design of non-clinical healthcare settings:

• Importance of human-scale spaces: avoid imposing architecture, particularly at entrances and reception areas.
• Sympathetic building form: embed the human preference for curvature, and craftsmanship within the quality design.
• Open layouts: diaphanous spaces with the flexibility to create enclosable areas, using high ceilings and sliding doors.
• Abundant natural light and air: use materials and designs that let light and air from fully glazed facades or extensive windows, smaller manually operable windows, clerestories, skylights, atria, courtyards, balconies or winter gardens, including adjustable shading devices.
• Warm materials: wood play a key role.
• Accessible landscapes: in- and outdoor spaces that include water features, and which are sensitive to seasonal and time changes.
• Warm spaces: include fireplaces and do not overheat (>24°C).
• Sensory spaces: focus on natural views, tranquillity, natural fragrances, sounds and textures.
• Colourful spaces: aiming for vibrant, high-contrast, quirky, colourful spaces.
• Vernacular marks: inclusion of local and traditional features strengthen a sense of belonging.

These conclusions were extracted from 474 direct quotations from Maggie's Centres users collected in these qualitative studies. Their comments were very rich and subjective in describing their experience and personal feelings about the spaces they use and their opportunities to connect with nature, which was extremely helpful to understand the role of biophilic interventions to elicit positive and pleasant feelings, sensations and thoughts. This list of conclusions represents just a synthesis of findings, and a more detailed discussion is presented in Section 3.

### 2.2. Study 2 outcomes

The systematically searched review followed rigorous replicable peer-reviewed steps and systematically identified nine studies that helped to identify and rank the biophilic design parameters that appear the most critical for promoting and supporting human health and well-being in clinical therapeutic environments, from the user's perspective. The results confirmed that biophilic design parameters in clinical environments cannot be examined under one umbrella concept, but the assessment should be specific to each type of space based on user groups: inpatients, outpatients and staff.

In terms of the ranking of biophilic design parameters, the order of importance was grouped mainly based on the results from Studies 1 and 3, however, Study 2 results contributed to the order of importance for some parameters. Particularly, Fresh Air and Thermal Comfort's rankings were increased in the final framework because they were highlighted in the studies examined.

The analysed findings in Study 2 considered the following general guidelines regarding the design of clinical healthcare settings, and supported the design guidelines in the framework proposed in this paper:

• Maximising biophilic parameters through the use of natural materials, natural colours, fresh airflow, natural light, safety and security.
• Windows design: afford views from clinical areas onto landscapes and the outside world, appropriate natural light exposure without glare, natural airflow and safety.
• Optimum stimulation: protect from overstimulation such as overpowering scents, noise, loud sounds, allergy-inducing and toxic plants, adverse comfort conditions and high-low temperatures coming from overexposure to the sun or shade.
• Providing a non-clinical feeling: by hiding medical equipment from the eye where possible, and aiming to create a homely, comfortable atmosphere.
• Offering accessible outdoor settings: that enable easy and effortless access, adequate greenery, comfortable amenities, balanced shade and sunny areas, porches, courtyards, patios, balconies, terraces and gardens.
• Facilitating socialising opportunities: through the spatial arrangement of seating and gathering options, the inclusion of communal spaces, children's play areas, semiprivate enclosures for personal conversations, and even BBQ areas.

These conclusions were extracted from user-focused quantitative and qualitative studies, which were systematically and rigorously selected in order to explore three main user groups' experience in clinical environments (in-patient, outpatient and staff) and their expectations from a healing healthcare environment. These results were synthesised from users' comments in 48 interviews and 1,827 surveys analysed in the examined studies. The outcome from this rich source of data was helpful to understand their priorities and the role that nature connectedness plays in therapeutic environments from their perspective. The findings from Study 2 are discussed in Section 3 in more detail.

### 2.3. Study 3 outcomes

**Figure 2:**
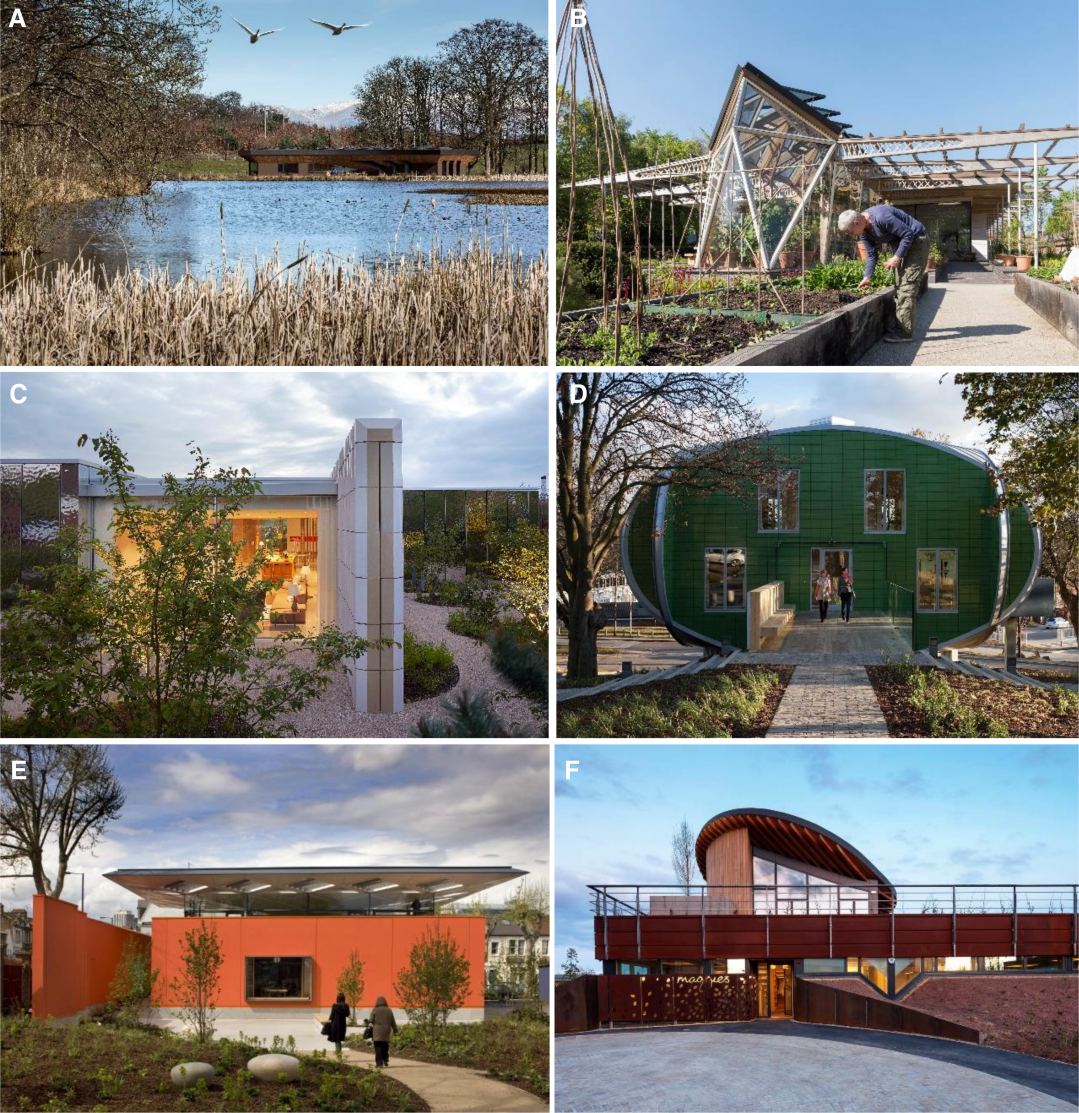
Views of the Maggie's Centres discussed in this study (**A**) Forth Valley located on the shore of Larbert Loch (Courtesy of Garber & James ©Keith Hunter), (**B**) Manchester (Courtesy of Nigel Young/Foster + Partners), (**C**) Southampton (Courtesy of AL_A ©Hufton+Crow), (**D**) Nottingham (Courtesy of CZWG Architects ©Martine Hamilton Knight ), (**E**) West London (Courtesy of RSHP ©Morley von Sternberg), (**F**) Newcastle (Courtesy of Cullinan Studio ©Paul Raftery).

The semi-structured interviews aimed to provide a means to contrast the results obtained from the systematically searched review and meta-synthesis studies presented in Studies 1 and 2 by crosschecking data obtained from a primary source, as well as to complement the input to the final conceptual framework based on recommendations offered by experts and practitioners. The interview population consisted of key stakeholders in the fields of psychology and architecture: Lesley Howells, psychologist and research lead of the Maggie's Centres Research Advisory Group (based in Maggie's Forth Valley); Darron Haylock from Foster + Partners (Maggie's Manchester, Circle Bath Hospital), Eoin O'dwyer from A_LA (Maggie's Southampton); Piers Gough from CZWG (Maggie's Nottingham); Lucy Brittain from Cullinan Studio (Maggie's Newcastle, The Catkin Centre and Sunflower House in Alder Hey Children's Hospital), and Ivan Harbour from RSHP (Maggie's West London, Guy's Cancer Centre)). The research reached saturation with these five architectural offices, as the designers of a selection of Maggie's Centres met a range of criteria set out to be covered in this study. This included: urban vs. rural settings; the use of low-key resources vs. non-restricted design; the employment of special materials; gender-friendly explorations and a variety of both early periods and recently designed centres. Figure 2 shows images from the Maggie's centres investigated in the interviews.

The questions designed for the architects were interested in generic design decisions, to start conversations that sought open answers to specific issues. These included issues such as assessment of their awareness of the biophilic design theory; Maggie's Architecture and Landscape Brief and its importance in the process; the effectiveness of communication and management with the client; the design process and steps that were unusual; design intentions, specifically in connection with considerations of the human-nature relationship in the spaces; the main design aspects or drivers behind the project; their background research and required consultant fields; their approach to site analysis/context in the project; their design considerations in relation to biophilic design parameters; the success of the buildings based on post-occupancy feedbacks and detected drawbacks; and environmental features that provided a healthy environment for patients and staff.

All interviews were recorded and transcribed verbatim. The transcripts were imported to NVivo 12 software for analysis, then coded and organised into exclusive and exhaustive categories to discover overarching themes, which are examined in the following sections.

The interview results provided a larger scale of information about therapeutic environments, as many of the architect participants were involved in the design of both a Maggie's Centre and clinical settings or hospitals. In the new conceptual framework, the most prominent contributions from Study 3 were that they clarified the importance of some parameters that appeared with a more ambiguous role in the existing literature (such as Water in non-clinical settings), offering a set of recommendations for decision-making and design process, and contributing to design recommendations to inform design practice in both clinical and non-clinical environments. Study 3 was particularly useful to shed light on the importance of specific stages in the design process, as well as clarify some parameters whose role could not be more precisely defined in Studies 1 and 2. Table 1 summarises an overview of the focus of the main comments and conclusions extracted from the interviews.

**Table 1:** Summary of the main design parameters extracted from the interviews.

		Brittain	Gough	Harbour	Haylock	O’dwyer	Howells
**Design Process and Decisions**	Maggie’s brief supported and encouraged	**✓**	**✓**	**✓**	**✓**	**✓**	**NA**
	Visiting the existing Maggie’s Centres	**✓**	**✓**	**✓**	**✓**	**✓**	**NA**
	Strong communication with the client	**✓**	**✓**	**✓**	**✓**	**✓**	**NA**
	Working with consultants and experts	**✓**	**✓**	**✓**	**✓**	**✓**	**NA**
	Selecting/transforming the site to maximise nature connection	**✓**	**✓**	**✓**	**✓**	**✓**	**NA**
	Budget friendly approach	** **	**✓**	** **	** **	** **	**NA**
	Male-friendly approach	**✓**	** **	** **	** **	** **	**NA**
**Prioritised Design Parameters**	Inclusion of greenery-plants	**✓**	**✓**	**✓**	**✓**	**✓**	**✓**
	Use of natural materials	** **	**✓**	** **	**✓**	**✓**	** **
	View of nature	**✓**	**✓**	** **	**✓**	**✓**	**✓**
	Existence of water elements	** **	** **	** **	** **	** **	**✓**
	Exposure to daylight	**✓**	**✓**	**✓**	**✓**	**✓**	**✓**
	Use of diverse colours	** **	**✓**	**✓**	** **	**✓**	**✓**
	Inside-Outside Connection	** **	**✓**	** **	**✓**	**✓**	**✓**
	Provision of multisensory environment	** **	** **	** **	**✓**	** **	**✓**
	Provision of thermal comfort variability	**✓**	** **	**✓**	** **	** **	** **
	Provision of welcoming-relaxing environment	**✓**	**✓**	**✓**	**✓**	**✓**	**✓**
	Provision of prospect	** **	**✓**	** **	** **	**✓**	** **
	Provision of refuge-feeling safe	**✓**	**✓**	** **	** **	**✓**	**✓**
	Provision of privacy	**✓**	** **	** **	** **	** **	**✓**

#### 2.3.1. Decision-Making and design process

The interviews provided insight into the decision-making and design process of a successful non-clinical environment. Firstly, a designer should do research and work with specialist consultants. Decision-makers, designers, management and administration must have knowledge about the importance of biophilic design so that decisions regarding the site, layout, building orientation, surrounding views, and so on can be considered in the planning stage. Skilled professionals need also to consider the repair and maintenance needs of biophilic features within available maintenance budgets. Secondly, visiting sites with a similar programme and located in a similar context is an important stage to detect problems and observe successful applications, and more importantly to know what the ambience is, how a day cycles, how they operate, and how people use the spaces. Designers should discuss with the user groups or conduct public opinion investigations to have more efficient human-centred buildings before setting up design drivers. If possible, they should select a place that offers access to natural processes, aim to approach the sites to maximise nature contact and offer a sense of enclosure from a highly urbanised and industrialised environment. Finally, site decision was claimed to be a critical part of the decision-making process. Decision-makers and designers should develop design strategies to enhance nature engagement and offer a connection with nature in all sensations even in naturally unfavourable environments. Natural passive design principles in terms of layout and orientation can be considered in a fixed position restricted site with activities and building works all around.

Although the priorities or level of importance for either type of setting differs for programme guidelines, spatial considerations and requirements in different therapeutic settings the recommendations stated above are applicable in all types of therapeutic environments. However, the design strategies and biophilic parameter requirements differ based on the user group and purpose of use.

#### 2.3.2. General comments on biophilic design parameters

Although all biophilic design parameters were not specifically discussed in the interviews by the interviewees, the most outstanding parameters were explained and referred to in the course of the conversations. This section indicates the application and perceived impact of these parameters.

Key design factors to achieve a relaxing atmosphere for patients were connected to design decisions related to materials, water elements, view of nature, nature immersion and bright environments. In particular, about creating a bright environment, the architects referred frequently to their consideration of daylight in the design and its benefits for health and wellbeing. The designs commonly employed big windows, skylights, reflective materials, and glass surfaces to be exposed to more daylight. Although a balance between light and shadow was aimed, the architects tend to increase openings considering the typically cloudy and rainy weather conditions of the UK. However, rather than maximising openings and glasses, Gough followed a more domestic approach by applying a Georgian-style window rhythm in the design, as he claimed that the best way of creating a balance between light-shade and thermal comfort is following the traditionally learnt way of local architecture, which also promotes a sense of belonging:

What is interesting is that Georgian houses have about an equal amount of windows and walls, and that produces very pleasant light in Georgian rooms. Terraced housing has windows where you get a bit of brick, a bit of window a bit of brick, a bit of window. That seems to be quite a nice balance of a sense of enclosure, good views, and the amount of daylight that you get. So this building was just built on that, it does not have too much light, it does not have too little, and it is balanced. And, of course, that is good for not overheating in summer and not losing too much heat in winter. So it is not a sort of all-glass extravaganza…Quite domestic.

In terms of providing a view of nature, the architects maximised the natural elements outside the building by designing gardens and landscapes to provide a direct view of natural elements through windows. Brittain, Gough, Haylock and O'dwyer claimed that they aimed to create a strong visual connection with the greenery on-site. Gough used a view of existing trees, while others produced their vegetation and landscape as the sites were located in a densely built environment.

**Figure 3:**
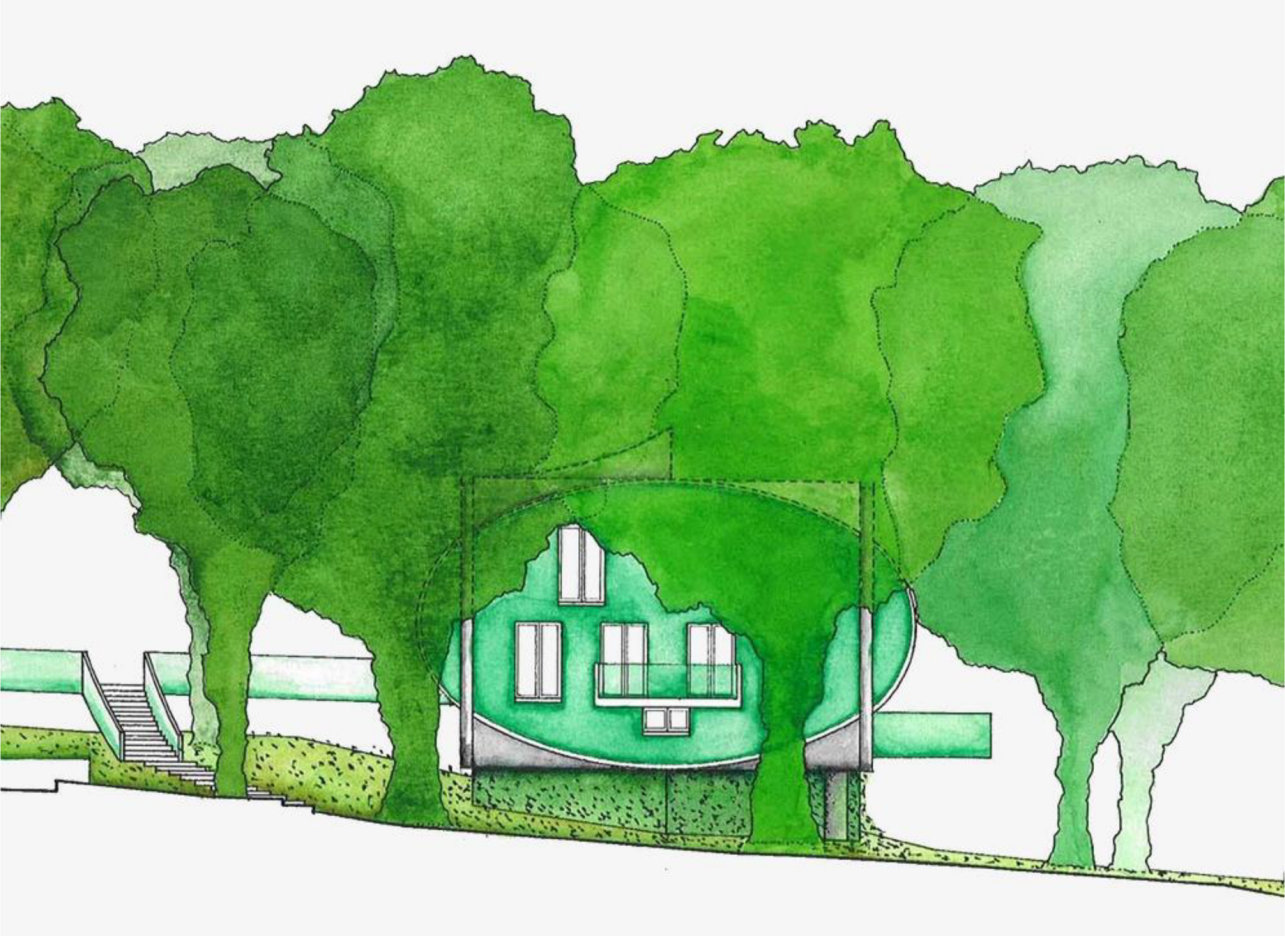
Visual connection with trees by lifting the building from ground level in Maggie's Nottingham (Courtesy of CZWG Architects ©Martine Hamilton Knight).

Gardens, plants and the connection between the building and landscape were one of the most outstanding aspects of Maggie's Centres design. All architects worked towards creating a strong connection with greenery and plants through different approaches. For example, in Maggie's Nottingham, where Gough created a strong visual connection with trees by lifting the centre from ground level and designing a garden and landscape around the building where the people can become involved with the planting and maintenance of the garden (Figure 3). He also warned that contact with nature, particularly the plants, should be in balance as nature is not beneficial every time based on his experience and knowledge:

The relationship between nature and people's feelings is definitely something to be looked at. So, there may be people who find too much of that. Too much of falling leaves might mean a bad time of year for feeling great about the world. But on balance, I am sure that it was the right decision to put [the centre] in this environment because it was so mature. And I think that is very comforting.

The connection between indoor and outdoor environments is an important feature of Maggie's Centres, according to the results of Study 1. The interviews confirmed that these connections were intentionally aimed by the architects. Howells emphasised that it is not easy in the UK to use outdoor settings in a planned way, as the weather conditions are unpredictable. However, architects were keen to create a connection with the outside in a more protected way. Big windows, doors, canopies, interior garden (courtyard), glass house etc. were successfully included in the designs. For example, the elevated structure of Maggie's Nottingham aimed to enhance the connection with the outside environment:

The main idea of the Maggie's was to put perch [sic] the building up in the tree canopy rather than down where the tree trunks are. You are kind of up with the branches. Then we have outside balconies coming out of the space. Not just look at the trees, but can almost shake hands with a tree because the branches are coming over onto the balcony, and you can sort of feel you are in them.

**Figure 4:**
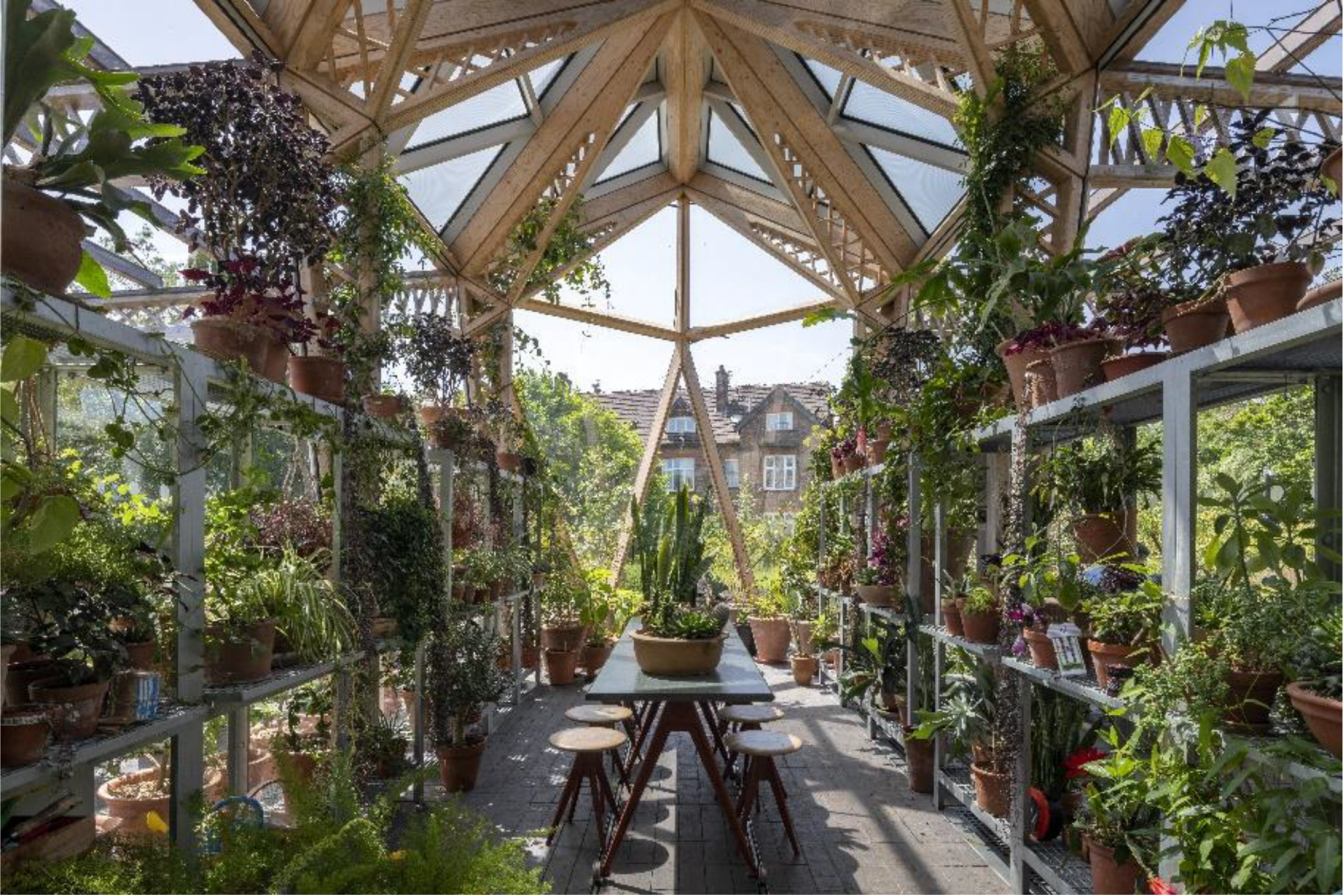
Winter garden in Maggie's Manchester (Courtesy of Nigel Young/Foster + Partners).

The architects aimed to create a multi-sensory environment mainly with vegetation, attracting wildlife, the smell of wood burning fire and water elements. However, as explained above, the gardens were the main source of multisensory stimulation, as Haylock defined the winter garden with its sensory characteristics (Figure 4): “the pillars of greenery and lushness and smells and a different environment…”

Although the importance of Water was not emphasised in Study 1, the interviews suggested that it should be ranked with a higher level of importance. Particularly, “a view of water” increases the quality of space. Described her observation and experience in relation to the view in Maggie's Forth Valley where the site is located beside a loch (Figure 2A). As a dynamic feature, the view of water offers a variety of views depending on the weather conditions and the time of day. Apart from the view and multisensory environment, the reflected light from the loch surface gives life to the building, and created a changing atmosphere experience in the centre:

If it is a sunny day, you end up with the reflection of the water cast into the centre. You can see the water or the ripples of the water, or the change of the light of the water playing out on one of the walls, in the interior walls. How lovely is that! I can walk around the corner, not looking towards the loch, I am looking into the internal wall, but I have got a kind of light playing on it. So I can just see the movement something that is outside. I do not know whether it was by design, but it is beautiful. It really is. There is life in this movement. And there is a sense of surprise. But in a good way. That is why when you walk in, you do not have to do something extra special, you just have some things which are a lovely surprise. The water playing on that wall, or the reflection, it is a surprise.

In terms of Fresh Air, the architects' statements proved the position reached in Study 1 as the air was the most underrated parameter in this study. Considering the vital importance of fresh air, it was likely that the importance of air was widely ignored by the participants as it was considered a given. However, Study 1 accepted that the air quality was sufficient in the centres because nobody reported any symptoms of the absence of fresh air. The interviews showed that the architects take great care of the natural ventilation of their designs. Moreover, Howells said that they have high air quality in the centres as they can easily ventilate inside, particularly after the COVID-19 pandemic, the need for ventilating the spaces increased:

After COVID times there is a lot of ventilation, so we use whatever ventilation is going on. There is a very discreet kind of vent then that we can open, then the doors. One of the things which are important in Maggie's Centre is the ability to let the outside in. So, all of the doors, for example, in this centre all the windows are French windows, so you can open the window as if it is a door.

**Figure 5:**
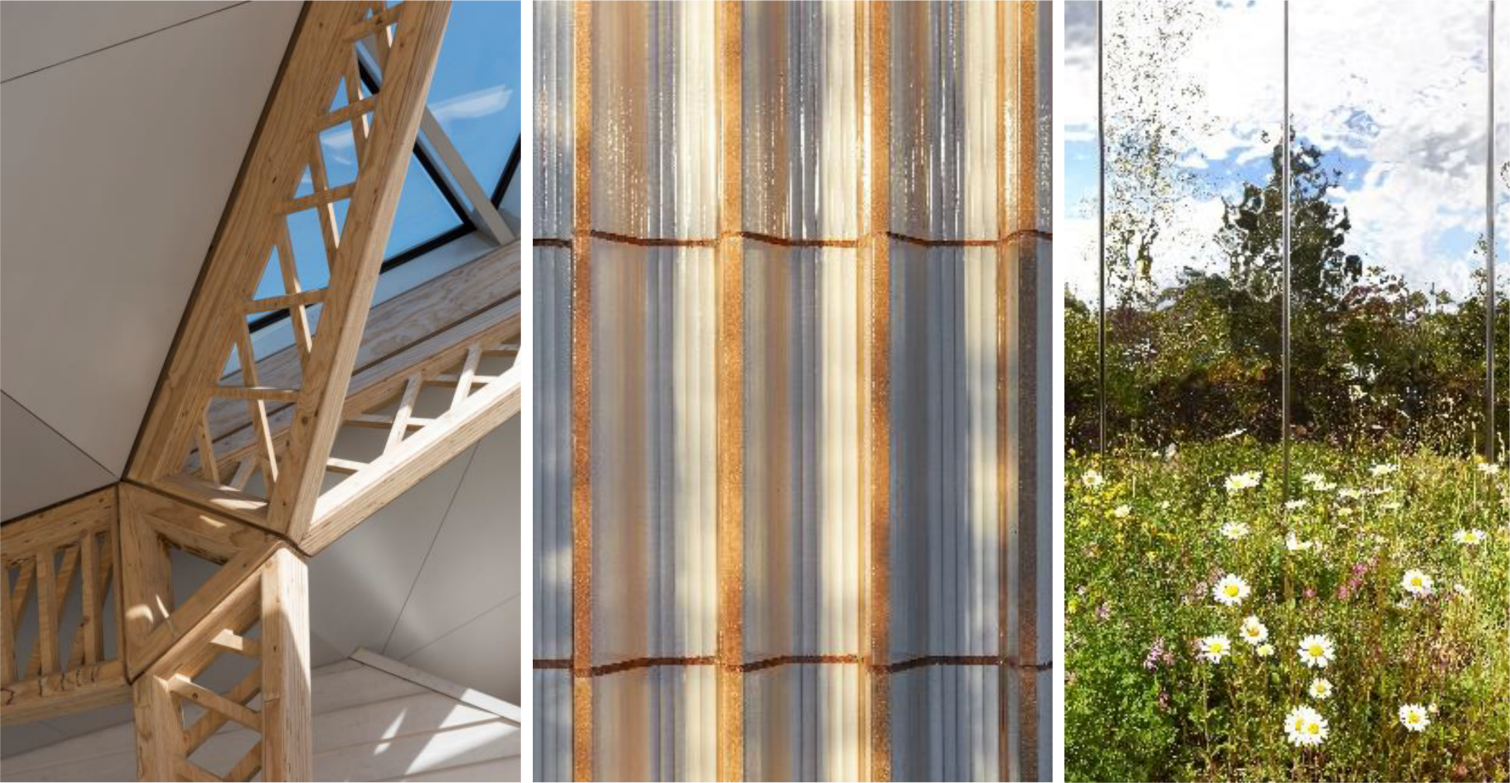
Timber trusses in Maggie's Manchester (Courtesy of Nigel Young/Foster + Partners), and ceramic walls and stainless-steel façade in Maggie's Southampton (Courtesy of AL_A ©Hufton+Crow).

Material choice was one of the most mentioned design vehicles to create a welcoming and relaxing environment by the architects. In particular natural materials, timber and ceramic materials were extensively preferred and recommended by the interviewees (Figure 5). Haylock stated that timber as the main material choice was mainly chosen to encourage a biophilic feeling:

The idea of using timber as a lightweight material, having that biophilic feeling was something that we borrowed from some early designs which was an aircraft hangar. How timber is used effectively, in a lightweight way, to create a light and airy structure.

O'dwyer highlighted ceramic material choices that encouraged and enhanced nature in space and the perception of contact with nature in Maggie's Southampton. Ceramic blocks were used in the construction of the walls instead of concrete. The earthy feeling of the ceramic facade was to endow a biophilic impression on the users:

The ceramic walls were quite rigid. But it worked really well against this very natural landscape. You have this kind of juxtaposition where you have a very strong form and a natural landscape working together. I think the choice of materials helps that. Also, you have got a reflective glaze on the ceramic, so it is flexible, a green landscape, but then you also have the earthy tones of terracotta clay.

Either using a more natural approach or a contemporary approach, architects employed colour as a tool to contribute to their goals. For example, Gough chose a green colour for his building, because it would be in harmony with the surrounding trees and green is the symbol colour of Nottingham. The striking red colour of Maggie's West London was aimed to stand out in contrast with the pale hospital campus, and thus attract people. Whereas in Maggie's Southampton, more earthy natural colours and clay were chosen in the ceramic walls, combined with pastel blue and pink tones to support wayfinding, as they used various colours in the walls for this purpose.

In terms of thermal comfort, the buildings aimed to get maximum sunlight as in the UK the solar gains for thermal comfort have to be maximised. Brittain explained their approach: “The building faces south to maximise solar gain, with solar shading to avoid overheating.” Howells also explained that all the centres employed operable windows, French balconies, etc. which also allows users to adjust the thermal comfort when they need.

Furthermore, the fireplaces help the architects to create thermal variability and comfort as well as entail a homely focus and the sensory experience contributed by burning wood as Harbour explained: “Maggie's said that we like to have a fireplace because it's a focus for home. Beyond that, I think, the warmness, comfort and smell of it [fire]…”

**Figure 6:**
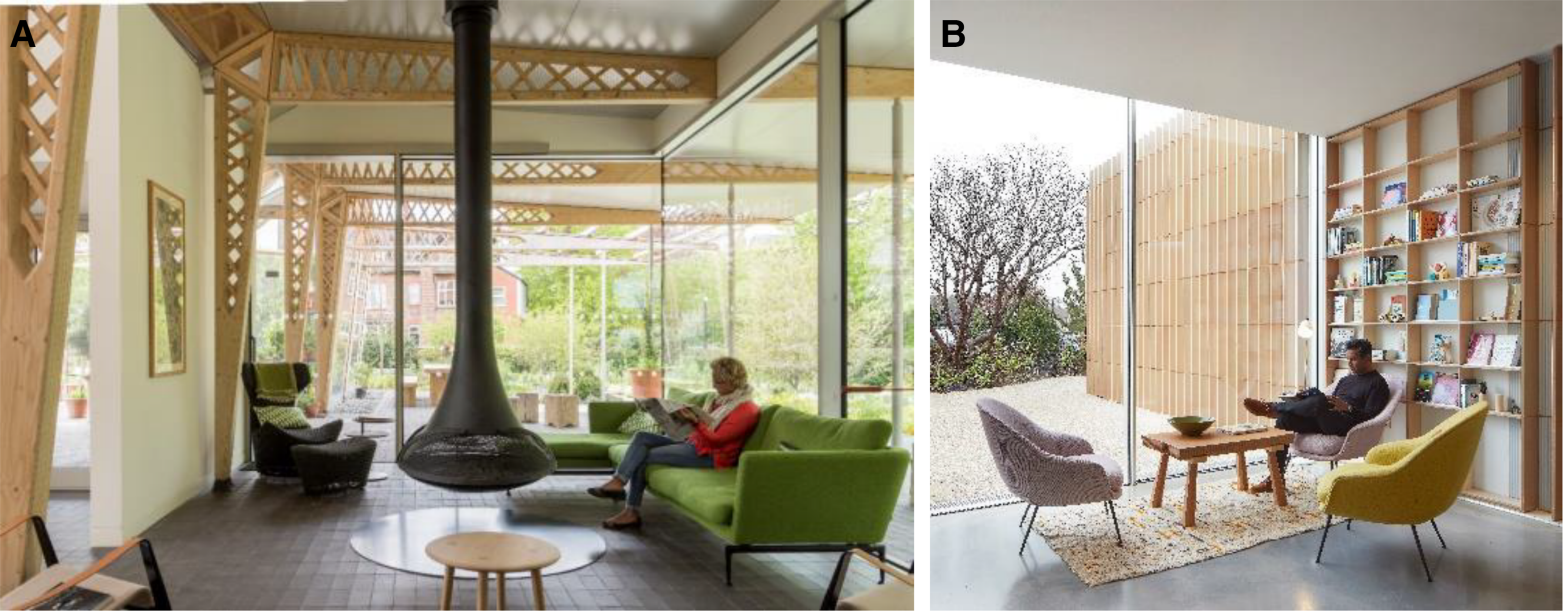
(**A**) Maggie's Manchester with homely furniture, wood-burning fireplace, glass façades, and timber components (Courtesy of Nigel Young/Foster + Partners), (B) Maggie's Southampton offers a bright environment with big windows (Courtesy of AL_A ©Hufton+Crow).

With the aim of creating a welcoming and relaxing environment, the architects' approach primarily tended to arrange a homely environment via comfortable furniture choices (Figure 6). The interviewees verified findings in Study 1 by emphasising not having a reception desk, kitchen with a family table, calming entrances, fireplaces, comfortable toilets, and not having signs in the centres enhanced the non-clinical and homely feeling.

Refuge, feeling safe and privacy were other important parameters the interviewees pointed out. The architects tried to create a refuge where the users were ensured with feeling safe. Using natural elements was quite common to reach this goal. For example, as mentioned before, Maggie's Southampton arranged landscape and vegetation to promote feeling safe, and Maggie's Nottingham was elevated from the ground level, like a treehouse, which also helped to create prospects and refuge. However, Gough claimed that although there is a prospect and refuge effect, it cannot be generalised for everybody as everybody is different and has different feelings about nature and feeling protected. Brittain and O'dwyer claimed that their key driver was to design the centres as an oasis where people take refuge and relaxed as the site is surrounded by the hospital environment:

Creating the building's own oasis was a key driver in the scheme—there was no relaxed outlook and we did not want the building to look back at the hospital so we created its own sheltered courtyard surrounded by mounded landscape. This provided a green therapeutic outlook with a calm courtyard which all the main spaces look out onto. It also benefits the surrounding buildings and car park by creating a green pocket in the hospital grounds.

**Figure 7:**
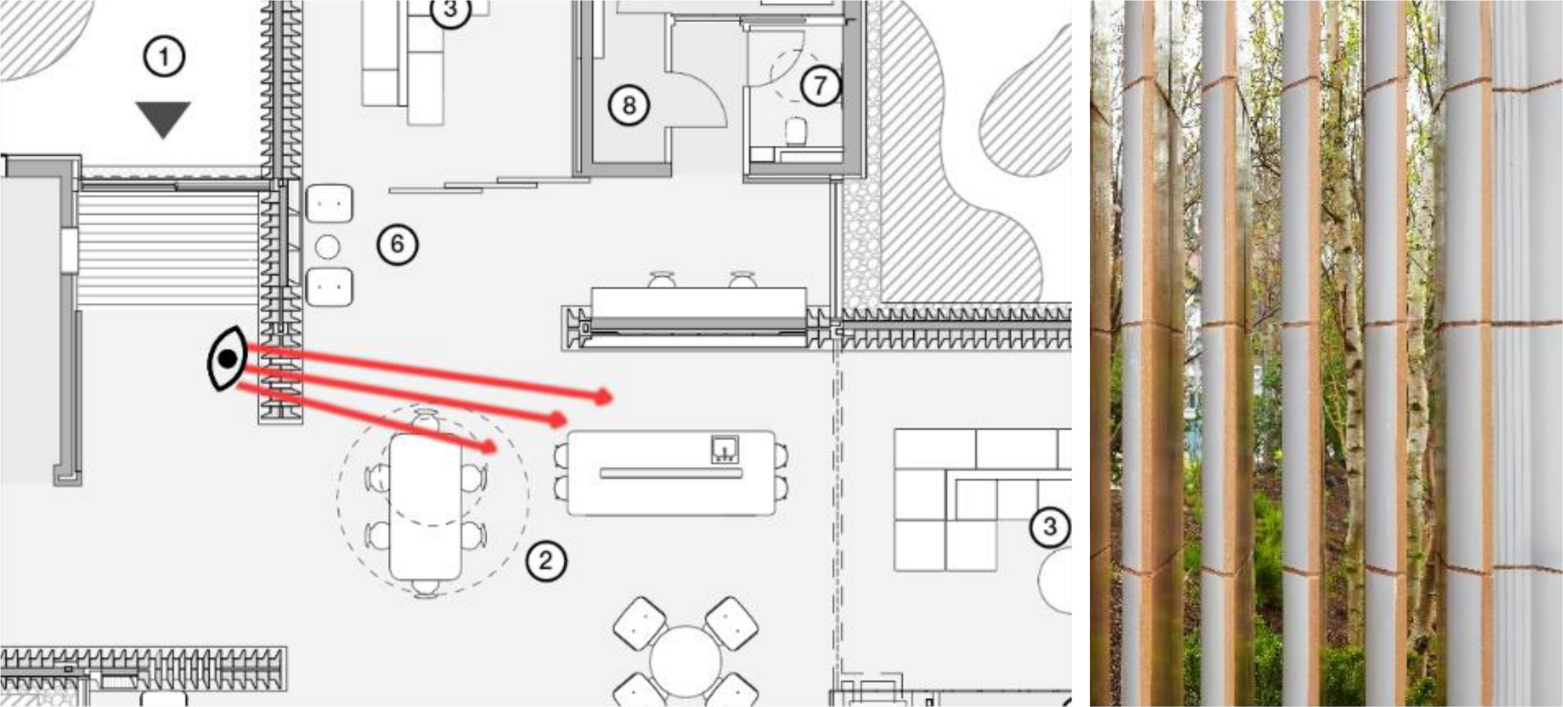
Maggie's Southampton: visual connection through the ceramic wall slits (©AL_A).

According to O'Dwyer, Maggie's Southampton's prospect and refuge approach at the entrance promoted a welcoming impact as they arranged the entrance lobby as a place where people can enter and pick leaflets to get information and see the kitchen through slits on the ceramic wall without being seen by the people inside, so they can decide to enter or leave without feeling any obligation (Figure 7).

Lastly, as privacy is considered an important parameter in the architects' statements, the post-occupancy evaluations also proved its importance. Online therapy from home during the COVID-19 lockdowns interestingly revealed the lack of an auditory sort of privacy opportunity that Maggie's Centres buildings normally provided:

So, I was talking about the idea of the change in ambient sound, which means that we cannot rely on ambient sound to create privacy in terms of conversations. So, it is tricky having these two conversations going on in the same space because you can overhear them. Whereas normally, you would not, because there would be enough chatter and there would be enough ambient sound. So that is one part where sound plays in. But the other part of it is that, particularly at the moment, households are very busy. If they have children, or if people are working from home, or you have got husband upstairs working from home, wife downstairs working from home, children at the kitchen table, everybody in the sitting room trying to work, then it is awful. So, Maggie's Centres have been a space of tranquillity as well. They are not comfortable and cannot speak openly because do not want to be overheard. They missed the most quietness and privacy in Maggie's Centre where they can talk about things that are potentially quite frightening. So yeah, that is definitely been something that we have been observing.

## 3. The framework and guidelines

**Figure 8:**
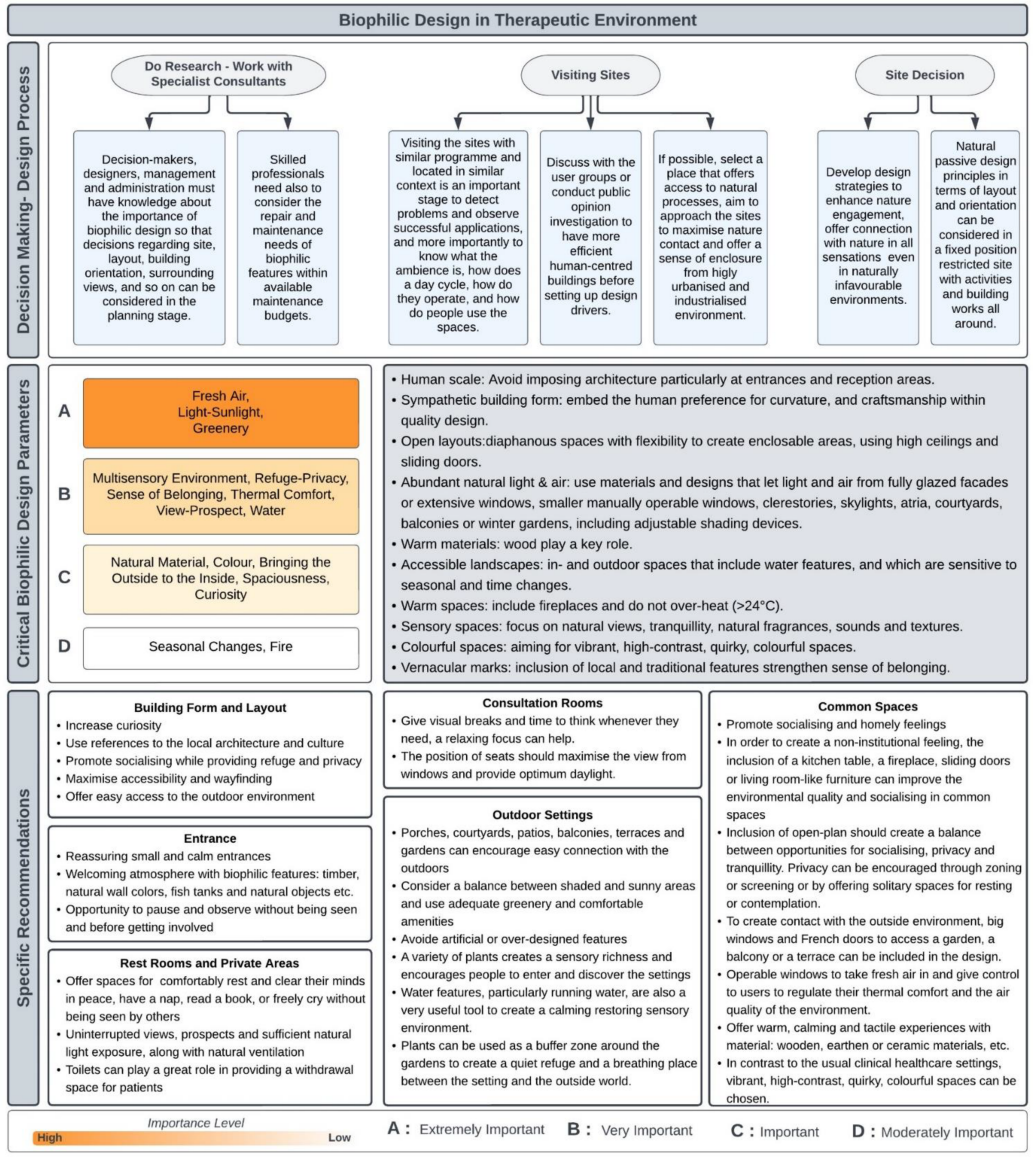
New conceptual framework for biophilic design in a therapeutic environments.

Synthesis of results from Studies 1, 2, and 3 are summarised within a holistic framework (Figure 8) that presents the analysis from all research methods in three different steps: the first part of the framework presents the recommendations for the decision-making and design process; the second step identifies and groups the critical biophilic design parameters; the last step conveys the summary of design recommendations revealed throughout the research.

The framework also aims to inform designers about the criteria that will make their designs biophilic. All parameters included in the framework are critical for the therapeutic environments, based on the findings reported in this research. Setting the order of importance for each group was mainly based on the results from the meta-synthesis (Study 1) and the interviews (Study 3), however, the systematically searched review results (Study 2) and the narrative literature review also affected the level of importance. For example, Fresh Air and Thermal Comfort's rankings were increased because they were highlighted in the studies examined as well as the current certifications (WELL certificate and Living Building Challenge). Thus, each of the four established levels of importance (groups A, B, C and D) will help to understand and apply the parameters in the design processes in a more efficient way. The order of importance shows that Group A (Fresh Air, Light-Sunlight, Greenery) represents *extremely important* parameters which are the most critical biophilic design parameters for users, therefore, a designer cannot claim a space as biophilic design if the space does not employ any of the parameters represented in this group. Group B (Multisensory Environment, Refuge-Privacy, Sense of Belonging, Thermal Comfort, View, Prospect, Water,) represents *very important* biophilic design parameters which are almost as important as the parameters in Group A. To create a biophilic space, designers should employ all the parameters in Group B rigorously, nevertheless, they can be disregarded only if experts prove that environmental conditions are unfeasible or the application of a parameter in this group can be harmful to some users. The parameters in Group C (Natural Material, Natural Colour, Bringing the Outside to the Inside, Spaciousness, Curiosity) are defined as *important*, and designers should employ these features as much as they can. Finally, Group D (Seasonal Changes, Fire) represents the *moderately important* parameters, but still, these parameters indicated their positive impact on the user's health and well-being, therefore, the inclusion of these parameters in a design will progressively increase environmental biophilic quality.

### 3.1. Design recommendations for implementing biophilic design parameters to create non-clinical therapeutic environments

As explained above, biophilic design can be efficient to support mental, emotional and physical health if the important parameters are combined in the space based on the users' needs. The following design recommendations bring together the findings of the three studies undertaken in this research, complemented by facts obtained from recent literature.

First of all, as summarised in Figure 8, designers should consider the following general guidelines regarding the design of non-clinical healthcare settings, namely: the use of human-scale spaces, sympathetic building forms, open layouts, abundant natural light and fresh air, natural warm materials, accessible landscapes, vernacular marks and warm, sensory and colourful spaces. To implement these general guidelines, based on examined literature on biophilic design and studies 1, 2, and 3, this study proposes following biophilic design application principles which represent the specific needs of this typology.

Primarily, a designer should prioritise working with real nature and natural elements, or at the very least simulated nature should be considered where the application of real one is not possible. Also, prioritising biodiversity and variability increases efficiency more than the quantity or area of natural elements.

The spatial organisation should allow users to exposure to natural views and multisensory natural environments for at least 20 min per day but no less than five minutes at a time (33). Thus, designers should consider how to enhance visual and non-visual connections in detail, such as user routes and circulation of the building that regularly passes across natural areas or arranging spatial layouts and furniture to provide uninterrupted view lines to natural landscapes in a seated position. Moreover, a simultaneous experience of visual and non-visual connection maximises the restorative quality of an environment.

As in nature, a biophilic design application should also reflect non-rhythmic stimulation on the senses, where the efficient frequency of non-rhythmic stimulation is about 20 s of exposure around every 20 min (33). The best way to create this atmosphere is to bring the outside to the inside and reflect seasonal changes in the space. For example, attracting wildlife (at least visually) through plants or fragrances, reflecting cloud movements or rain, taking a breeze in, or exposing the building to spontaneous natural sounds such as birds chirping or water babbling.

In order to create contact with the outside environment, big windows and French doors to access a garden, a balcony or a terrace can be included in the design. Barriers between the outside and the inside should be removed as much as possible. The most noticeable factor of the inside-outside relationship should be the affordance of visual connection, such as a view of the sky, water or greenery. As some patients do not have the energy to walk around, they should find an opportunity to have access to nature while being inside. Windows should be operable with the aim to take fresh air in and give control to users to regulate their thermal comfort and the air quality of the environment. (Studies 1, 2, and 3).

Thermal variability is another stimulating feature in design as the temperature changes non-rhythmically in nature, providing thermal variability in space will increase comfort and perception, however, overstimulation should be avoided. To distribute and prolong thermal variability, designers can incorporate other biophilic design parameters (e.g., fresh air flow, daylight, natural materials) or mechanical and electronic systems can be applicable where necessary. In order to provide healthy thermal comfort, the temperature should be between 18°C and 24°C (39, 40). Even though the users are given the option to control the thermal quality of the space, designers must avoid reaching a temperature of 24°C to not remind a hospital ward which creates an unwelcoming feeling. Designers can avoid temperatures over 22°C where it is safe for users to create a more welcoming and relaxing space. Therefore, operable windows are strongly recommended with the aim to take fresh air in and give control to users to regulate their thermal comfort and the air quality of the environment (Studies 1, 2, and 3). Since an efficient biophilic design considers fresh air level rigorously, architectural elements for natural ventilation should be prioritised over mechanical ventilation where possible. Therefore, to improve indoor air quality and provide a better state of health, ventilation rates should be higher than 20 cfm and up to 40 cfm per person in space (41, 42).

In terms of greenery and plants in biophilic design, supporting evidence suggested that a high density of plants in an indoor environment also decreases cognitive performance as well as the quality of space (43). Therefore, a moderate amount of greenery should be engaged based on the spatial programme. The general concept of biophilia claims that the application of single or isolated plants is not effectively beneficial. Vegetation should be rich and ecologically connected while the plants should be chosen from local species. Although designers should prioritise local plants and vegetation, it should be taken into account that slightly scented plants with green and small leaves are the most appropriate and effective plants for health and well-being, whereas red flowers produce a fatiguing impact over time (25, 44, 45).

Although one of the key design factors to achieve a relaxing atmosphere for patients was the inclusion of water elements, wrongly implementing them can cause discomfort. Repetitive and abundant experience with water can cause a loss of interest. Moreover, a high volume of running water can reduce the acoustic quality of the space and increase humidity (46). Hence, an optimum amount of water features should be implemented in practice, avoiding exaggeration. Also, the restorative effect of water depends on its quality. Clear water should be prioritised and designers should also consider the sustainability of water quality and its maintenance, as dirty and brown water is less restorative than clear water (47–49).

A healthy environment provides an opportunity for direct exposure to sunlight (approximately 3,000 lux) for at least 30 min a day (50). When designing lighting and taking daylight inside, it is critical to consider a balance between dynamic and diffuse light to avoid a negative impact. For example, long-time direct sunlight penetration, changing light colours or sharp transitions can create discomfort. Consideration of circadian lighting is also critical (51–54), particularly in long-period occupied spaces. Therefore, various architectural elements can be used to get more daylight inside, such as clerestories, roof fenestrations fitted with selective shading devices, roof openings, atria, courtyards, glass-walled porches, and small openings and skylights. The amount of daylight and ceiling height greatly improves the quality of space and the perception of spaciousness, which helps to stop claustrophobia and to reduce the feeling of stress. Also, spaciousness with the explosion of volume can be used for triggering curiosity and a welcoming feeling (Studies 1, 2, and 3). Additionally, daylight can be a tool for creating a distinction from the usual healthcare facilities, along with the direct benefits of daylight. Since the daytime is quite short in winter (in the UK), artificial lighting should be designed in accordance with the natural light spectrums. This study showed that the warmth of soft light was associated with feeling safe, thus, the artificial lighting used in the buildings can be chosen to be within the warm (3,000–4,000 Kelvin) or soft (2,700–3,000 Kelvin) range. The lighting should be designed specifically in some rooms, for example, the art therapy classes require bright light, while softer and dimmer light is used in relaxation classes in some of Maggie's centres (Studies 1 and 3).

Since human receptors can detect and differentiate real and synthetic materials, real natural materials would be more effective and stimulating. According to studies on timber, the application of wooden materials on 45 per cent of the whole surface creates a feeling of comfort, and over-use can cause harm to cognitive performance (55). Thus, designers should avoid monotonous overstimulating natural material applications and can use various materials to buffer and soften overstimulating or boring atmospheres. According to Studies 1 and 3, the material choice should offer warm, calming and tactile experiences. For example, wooden, earthen or ceramic materials can be employed in construction. Material choice, organic shapes, and structural elements can be used to attract attention in settings since a visual focus or distraction helps some patients to forget their unpleasant thoughts. However, concrete or steel like “cold” materials should be softened by combining them with natural materials or paint. Strategic material craftsmanship and structural components can be used to arouse curiosity and invite people to explore the setting, particularly to attract more men visitors as observed in the study. In any case, the surface materials should be warm to the touch, and plastic materials should be avoided (Studies 1 and 3).

Likewise, the colour choice should follow the same principles to avoid a feeling of dullness. The designers should aim for vibrant, high-contrast, quirky, colourful spaces in contrast to the usual clinical healthcare settings. According to the analysis in this research, colourful decoration gives a sense of family and floods people with feelings of welcome and relaxation. Moreover, different spaces with different colours help people to look from a different perspective (Study 1). Moreover, various colours impact human psychology in different ways: soft and natural blues help to feel relaxed as they remind us of the sky and water; shades of vibrant green give energy and make people calm as they are associated with meadows or forests; yellows are warm and welcoming and create a social and energised atmosphere as they remind us of warm summers and the sun; purple and mauves are spiritual and meditative colours, and evoke mystery as they represent dawn and dusk; oranges and reds can be energising, exciting and stimulating as they are the colours of ripe fruits and berries; dark colours are associated with sophistication, depth and mystery, and feelings of security and refuge, but if they are not used carefully the space can easily be oppressive and overwhelming (56). With this in mind, using colour in much the same proportions and with a sense of harmony as in nature, is an important point to avoid overwhelming people.

Within the setting, designers can use vernacular elements from material to furniture choice. The inclusion of local and traditional architectural traces and elements from the “home” culture of the local users will strengthen a sense of familiarity and a sense of belonging, which contributes to welcoming and relaxing feelings. In order to create a homely environment, the designers should understand very well the local people's own perception of what home means, as they will be the main user group and the notion of family and home culture depends on the context in which they grew up. Besides, nature-based smells and sounds should be used while avoiding chemical medicine-like fragrances and sounds, because the multi-sensory experience has a striking impact to help people to create a connection between their memories and space. It should be taken into consideration that, as it was clearly detected in this study, combining both familiar and relaxing domestic features that make people feel safe and homely with surprising and stimulating features will attract people by curiosity (Studies 1 and 3).

Lastly, the space should offer a sense of protection and refuge, as the user groups will mainly be vulnerable patients or their relatives. Façade openings can be designed following the prospect-refuge principles, in which the main idea is “see without being seen” (57). Low-level refuge and high-level prospect combinations were found to be restorative, whereas low-level prospect and high-level refuge can increase stress, fatigue and negative emotions (58). Therefore, a moderate prospect distance should be higher than six meters (short depth), although the distance of the preferred prospect was stated as above 30 meters (long depth) (59). A prospect can be applicable in both interior and exterior spaces; the interior prospect is to provide a visual connection between the spaces and it has a greater impact with the opportunity to see multiple spaces together. Prospect and refuge can be designed and regulated by orienting buildings, corridors, glass walls, or playing with ceiling heights. Also, a refuge space might be created through the use of light and shadow, which can also endow a mysterious character to the space. View angles can be arranged in this regard, and be supported with greenery and plants in the garden. Screening on some windows or curtain systems can also be implemented (Studies 1 and 3).

Apart from the general guidelines for biophilic design parameters in non-clinical therapeutic environments, this study identified space-based design recommendations for different parts of the non-clinical setting design according to two different user groups: patients and staff. The next section presents the design recommendations more specifically for these two groups.

### 3.2. Space-based recommendations for non-clinical settings from a patient-centred perspective

Although the existing frameworks reported recommendations for each biophilic design parameter in a general sense, this research also examined the space-based application of biophilic design, which is a novel approach in this field. Based on the analysis of Studies 1, 2 and 3, various biophilic design recommendations have been proposed for different parts of the non-clinical settings as well as the buildings' form and layout design.

#### 3.2.1. Building form and layout

There are two considerably successful design strategies found in terms of the use of building form. Firstly, as a tactic to elicit curiosity in order to attract people to the centre. However, these unfamiliar forms can sometimes prevent them from providing a homely human-scaled environment, thus, the architects should select their drivers rigorously and approach unfamiliar forms well thought throughout. Secondly, as a way to create a sense of belonging for the visitors, designers can follow the trail of local architecture and culture (Studies 1 and 3).

In terms of layout design, it should encourage the visitors to socialise while providing an opportunity to withdraw when they need it. The visual connection between the different parts of the buildings is also another necessary contributor to enhancing welcoming and relaxing feelings. Thus, an open-plan approach was the most commonly preferred strategy for layout, as it can also promote a non-clinical feeling. However, this visual and social connection should also be in balance with the needs of staff who sometimes need to be away from the visitors for their personal work and have some breaks (Studies 1,2, and 3).

According to cancer patients' preferences identified in Study 2, ease of movement is one of the most important aspects that the buildings should offer to patients. As such, the maximisation of accessibility and the removal of barriers should be seriously considered. This includes rapid and easy access between outdoor settings, foyer-waiting rooms and treatment settings with safety that must be considered as an overarching priority in relation to movement. For example, the use of non-slip surface materials, smooth paved paths, ramps rather than steps and colour-contrasting curbing along pathways. Barriers to be avoided can be heavy doors, narrow doorways and pathways, etc. In order to provide physical access to the outside, all barriers and thresholds should be removed for patients. In some cases, automatic doors can be suggested to improve ease of access.

The material choice and heating system are another concern in terms of the thermal comfort of the patients, who are usually more sensitive as a result of chemotherapy, as it was reported that the environment often tended to be over-hot in hospitals (Study 2). Also, as was reported by users in Study 1, the material quality and features are more important than the design or price of the furniture. Therefore, plastic materials should be avoided as furniture options as it increases temperature perception.

#### 3.2.2. Entrance

The entrance to the facility is an important space, as upon arrival people often face high levels of stress and anxiety. Therefore, creating a welcoming atmosphere with biophilic touches can relax people: for instance, with the use of natural materials such as wood, natural wall colours, fish tanks and natural objects. As always, safety should be a paramount design criterion, avoiding the inclusion of allergy-inducing elements, and slippery or otherwise challenging surfaces (Studies 1, 2, and 3).

Reassuring small and calm entrances can encourage people to enter the building. As these non-clinical environments are envisioned to be environments where the visitors receive mental, psychological and social support, entering the building usually means that they have accepted their illness and decided to fight it, which is a turning point for the visitors. Therefore, the entrance is a space that should be distinguished in its design. Curiosity or familiarity (that promotes a sense of belonging) can be applicable principles in distinguishing the entrances. As learnt from Maggie's Centres, not having a reception desk creates a homelier character and less institutional atmosphere, which also contributes to social interaction among the visitors (Studies 1 and 3).

Along with the physical interventions, prospect and refuge should also be considered specific to the design of entrances, where the users should have the opportunity to pause and observe without being seen and before getting involved in any activities and decide to participate without feeling pressure or obligation (Study 3).

#### 3.2.3. Rest rooms and private areas

Therapeutic environments also offer spaces for users to retreat and rest in more private corners or rooms where they can comfortably rest and clear their minds in peace, have a nap, read a book, or freely cry without being seen by others. Study 2 revealed that connection with the outside and nature is highly demanded in these kinds of more private spaces. Learning from inpatient environments for cancer patients, windows should provide uninterrupted views, prospects and sufficient natural light exposure, along with natural ventilation. Supporting evidence suggested that approximately 300 lux daylight is sufficient in inpatient rooms (60), thus, this amount of daylight can also be adaptable in these non-clinical private spaces. Window design should also pay attention to privacy, safety and refuge by providing one-way views. Indoor seats or beds that are strategically located to maximise the use of natural window views can motivate patients to take advantage of these opportunities (Study 2).

As observed in Maggie's centres, toilets can play a great role in providing a withdrawal space for patients, they can offer a more spacious atmosphere where patients can have solitary breaks or comfort to cry freely (Studies 1 and 3). Study 2 also revealed that toilet entrances should be protected from others' sight since some cancer patients need to use toilets more frequently and some reported that they do not want to be seen always waiting for the toilet.

#### 3.2.4. Common spaces

As Maggie's Centres are the case study for non-clinical settings in this research, it is important to understand their kitchen concept, as it provides the core of the communal space in Maggie's Centres. In the kitchens, the table was the most distinguishable characteristic that promoted socialising and homely feelings. Thus, the common spaces should have comfortable, relaxing, and socialising characteristics as well as provide features that promote refuge and feeling safe. In order to create a non-institutional feeling, the inclusion of a kitchen table, a fireplace, or living room-like furniture can improve the environmental quality in common spaces, as all of them also contribute to socialising. It was particularly noticed in this study that providing a fireplace in these centres, with the smell of burning wood, the crunching sound of it, and the visual and thermal effect of fire itself, was a prominently effective tool to restore the quality of the space (Studies 1 and 3). As explained before, an open-plan layout provides the highest exposure to daylight and socialising opportunities, but it also creates a noisier environment and impacts the provision of withdrawal spaces. Therefore, the inclusion of open-plan spaces needs more thought in order to create a balance between opportunities for socialising, privacy and tranquillity. In common spaces, privacy can be encouraged through zoning or screening or by offering solitary spaces for resting or contemplation (Study 2). Moreover, sliding doors are preferable, as it was found that sliding doors promote a feeling of relaxation and privacy. Also, they contribute to the non-institutional feeling, along with the notion of “signlessness”, in which the settings decide not to use any sign on the doors (Studies 1 and 3).

#### 3.2.5. Consultation rooms

The consultation rooms are the places where direct psychological therapy is delivered. The position of seats should maximise the view from windows to allow patients and psychologists to give visual breaks and time to think whenever they need since the topic sometimes can be intense and they might need a relaxing focus. These rooms work in a kind of similar way to the specialist care units investigated in Study 2, therefore, seats near the window were also regarded there as the most preferred location within the treatment rooms, in which optimum daylight and uninterrupted views for a larger portion of the room were sought. Moreover, as learnt from Study 2, cancer patients seek a spacious calm atmosphere while consulting with a doctor, nurse, or specialist.

#### 3.2.6. Outdoor settings

The importance of easy and effortless physical access to outdoor environments is frequently emphasised in Study 2. Thus, porches, courtyards, patios, balconies, terraces and gardens can encourage easy connection with the outdoors. However, it is important to consider a balance between shaded and sunny areas and use adequate greenery and comfortable amenities.

Garden design should reflect nature by avoiding artificial or over-designed features. The variety of plants creates a sensory richness and encourages people to enter and discover the settings. Also, various plants reflect seasonal changes and transform the atmosphere every day. Gardens should be enriched with diverse plants and flowers to heighten and uplift the senses. Also, wilderness such as birds, bees, or small animals, can be attracted or owned to trigger all senses: the smell of scented plants and blossoms, the tactile texture of the tree trunks and sitting on the grass, hearing rustling leaves and rain's pattern on the leaves, hearing the singing of birds that perched on the trees and chickens crowing, or the taste of edible plants and fruits and so on. Water features, particularly running water, are also a very useful tool to create a calming restoring sensory environment. A well-designed pool or fountain can easily promote the environmental quality of the gardens (Studies 1 and 3).

Moreover, in an urban context, plants can be used as a buffer zone around the gardens to create a quiet refuge and a breathing place between the setting and the outside world (Study 1).

A glass house or winter garden concept can be integrated into the setting, which can become a distinctive characteristic of the centre, where the users can enjoy diverse vegetation in any season and a multisensory environment and are involved in activities to grow plants. This concept can help to improve spatial and biophilic quality as they offer easy access to natural elements to users, particularly those who do not have enough power to walk out, in all seasons. Regarding patients who might be sensitive to cold weather, this kind of sheltered space can confidently offer contact with natural features such as daylight, fresh air, greenery, and a multi-sensory environment. Additionally, the production of plants and vegetation in these greenhouse-like spaces can also contribute to the setting's social opportunities (Studies 1 and 3).

Lastly, experts supposed that including physical exercise opportunities (regarding patients' physical ability) can also be helpful for their mental states such as stroll gardens, walking paths, meandering trails and resting points, mobility and balance training, gardening tasks, assisted walking and labyrinths (Study 2).

### 3.3. Design considerations from a staff-centred perspective

In addition to the patient-centred perspective for designing non-clinical settings, some special design considerations for staff emerged in the Studies, particularly for the staff break areas.

A homely environment is recommended in break areas and their offices (or consultation rooms), where a sensorial connection with nature could provide a relaxing environment to reduce stress. The furniture in staff areas should be easily rearrangeable, and comfortable, for individual and group activities, with sofas and recliners (Study 2). View through windows is a frequently desired feature within staff indoor break areas, since visual or physical contact with the outside world and biophilic elements (e.g., View, Prospect, Daylight) played a critical role in staff's restoration. In fact, the most powerful stress reliever was found to be the provision of direct access to the outdoors, because of the opportunities to direct contact with natural elements (Studies 2 and 3).

The need for privacy was identified clearly by Maggie's centres' staff, as privacy for staff is crucial for their comfort, at least for their private meetings or phone calls (Studies 1 and 3). Although staff want to have private spaces, they still strictly indicated that the best withdrawal space should allow a one-way visual connection with patients to keep an eye on them, thus, they can rest and relax comfortably (Studies 1, 2, and 3). Given that refuge, privacy and quietness are the most important biophilic parameters for staff, designers may think of private outdoor break areas free from patients and their companions where the environment is enriched with greenery, trees, shade, tables, flowers and water features. Providing private outdoor break areas would increase the speed of refreshing during the breaks (Studies 1, 2, and 3). However, these staff break areas should be located in ways that provide easy and rapid access back to patients (Study 2).

## 4. Conclusion

In comparison to the previous frameworks, this study presented a biophilic design framework specifically developed for therapeutic environments and specific to non-clinical typologies in the UK context (Table 2). The criterion for biophilic buildings was clearly stated by hierarchising biophilic design parameters based on the users' requirements as presented in Figure 8. Therefore, the new conceptual framework directs designers and more precisely draws the borders of biophilic design, in contrast with some practice that uses biophilic design as a self-promoting tool, employing an insufficient and inefficient application of biophilic design parameters. The new framework was also supported by design recommendations from general principles to specific recommendations for spaces in therapeutic environments that will guide designers to fulfil required biophilic design features.

**Table 2:** Comparison of the existing biophilic design frameworks and the new conceptual framework.

Existing Biophilic Design Frameworks and Guidelines				The New Conceptual Framework for Biophilic Design in Therapeutic Environments
Dimensions, Elements and Attributes of Biophilic Design	The Practice of Biophilic Design	14 Patterns of Biophilic Design	Nature Inside: A Biophilic Design Guideline	
General to all typologies	General to all typologies	General to all typologies	General to all typologies but with examples of different typologies	Specific to non-clinical therapeutic environments
Unnecessary categorical division and insufficient definition of parameters	Adequate definition of the parameters	Adequate definition of the parameters	No definition of the parameters	Adequate definition of the parameters
Slightly supported by scientific knowledge	Moderately supported by scientific knowledge	Supported by scientific knowledge	Moderately supported by scientific knowledge	Fully Supported by scientific knowledge
No reference to cultural or regional characteristics	No reference to cultural or regional characteristics	No reference to cultural or regional characteristics	No reference to cultural or regional characteristics	Based on the UK context: Western culture and humid temperate climate
No indications of an order of importance for parameters	No indications of an order of importance for parameters	No indications of an order of importance for parameters	No indications of an order of importance for parameters	Hierarchised and standardised recommendations, based on order of importance in the use of parameters
Very rarely included guidelines for practice	Occasionally included guidelines for practice	Included general guidelines for practice	Included guidelines for practice based on specific examples	Included detailed general and specific guidelines for therapeutic environment practice

Moreover, this study proposed a guide for biophilic design applications in non-clinical environments with a particular focus on cancer patients. The guidelines were mainly shaped around the needs and problems of cancer patients. The recommendations addressed their physiological needs (i.e., recommendations regarded various side effects such as sensitivity to cold or smell by offering patients control over the thermal variability or fresh air, access to sunlight, furniture with natural material to prevent over-heat etc.) and psychological needs (socialising opportunities, more private refuge spaces, relaxing and calming indoor and outdoor spaces with natural elements, offering visual focuses with nature view or daylight to distract unpleasant thoughts etc.). Although the existing regulations and standards (61–65) emphasised the importance of natural light, view, fresh air, thermal comfort, access to natural spaces and privacy, they do not specialise in biophilic design and specific populations, and they do not indicate biophilic parameters directly but use biophilic values as criteria among the many other non-biophilic features. On the other hand, the guidance in this study proposes a clear frame for biophilic design applications (in which designers know how to classify their spaces as biophilic), in a specific typology (non-clinical therapeutic environments) for a specific population (those affected by cancer) in a specific climate (humid temperate oceanic climate) and specific cultural context (western culture in the UK) in accordance with our position to the biophilic design's definition.

To sum up, based on our findings in these studies this research proposes a new definition of biophilic design that will reduce misunderstandings in practice, application of regulations and research environments: biophilic design is a harmonious application of natural parameters that works together in order to make the users feel connected to nature holistically. All biophilic design parameters cannot be equally important for every type of building, this harmony should be specifically established for each building programme and the particular type of climate and local culture in which the building sits, since people have different notions and perceptions of nature.

Future research should also develop a biophilic design framework for clinical environments based on the types of illnesses (even on the types of cancer), side effects, environmental perception of patients, and biomarkers changes. However, this kind of research needs an interdisciplinary team including experts in a variety of fields such as architecture, medicine, and environmental psychology. Moreover, future research should investigate the different building typologies and programs based on their specific user groups and contexts in various regions, climates and cultures which is necessary to contribute toward more effective localised biophilic design frameworks.

## Data Availability

The original contributions presented in the study are included in the article, further inquiries can be directed to the corresponding author.
